# Nerve-Sparing Robotic-Assisted Radical Prostatectomy Based on the Absence of Prostate Imaging-Reporting and Data System ≥3 or Biopsy Gleason Pattern ≥4 in the Peripheral Zone

**DOI:** 10.3390/cancers17060962

**Published:** 2025-03-12

**Authors:** Yoichiro Tohi, Hiroyuki Tsunemori, Kengo Fujiwara, Takuma Kato, Kana Kohashiguchi, Asuka Kaji, Satoshi Harada, Yohei Abe, Hirohito Naito, Homare Okazoe, Rikiya Taoka, Nobufumi Ueda, Mikio Sugimoto

**Affiliations:** Department of Urology, Faculty of Medicine, Kagawa University, Takamatsu 761-0793, Kagawa, Japannaito.hirohito@kagawa-u.ac.jp (H.N.);

**Keywords:** nerve-sparing, PI-RADS, prostate cancer, resection margin, robot-assisted radical prostatectomy

## Abstract

**Background/Objectives**: The objective of this study was to evaluate the oncological outcomes and safety of nerve-sparing (NS) robot-assisted radical prostatectomy (RARP) when applied without Prostate Imaging-Reporting and Data System (PI-RADS) ≥3 lesions or Gleason pattern ≥4 on biopsy in the peripheral zone (PZ). **Methods**: We retrospectively analyzed 208 patients who underwent RARP between August 2017 and December 2022, excluding those who had received preoperative hormonal therapy. After NS status stratification and patient characteristic adjustment using propensity score matching (PSM), positive resection margin (RM) rates and prostate-specific antigen (PSA) recurrence-free survival were compared. Urinary and sexual quality of life (QOL) were assessed using the Expanded Prostate Cancer Index Composite, along with predictive factors associated with positive RM and RM locations in the NS group. **Results**: NS was performed in 68.6% (*n* = 129) patients. After PSM, there were no significant differences in RM positivity (*p* = 0.811) or PSA recurrence-free survival (Log-rank *p* = 0.79), regardless of NS status. There was no difference in sexual function between groups, but urinary QOL was significantly better in the NS group from the third month onward. In the NS group, RM positivity was 27.9% (n = 36), and diagnostic PSA (odds ratio [OR], 1.110, *p* = 0.038) and clinical T stage (OR, 1.400, *p* = 0.038) were predictive factors. The RM positivity rate on the NS side was 10.8%. **Conclusions**: NS, based on the absence of PI-RADS ≥3 lesions or Gleason pattern ≥4 in PZ, did not increase RM positivity rate and increased early urinary QOL.

## Simple Summary

This study evaluates the safety and oncological outcomes of nerve-sparing (NS) robot-assisted radical prostatectomy (RARP) when performed only in patients without Prostate Imaging-Reporting and Data System (PI-RADS) ≥3 lesions or Gleason pattern ≥4 in the peripheral zone (PZ) on biopsy. A retrospective analysis of 208 patients undergoing RARP between August 2017 and December 2022 was conducted, excluding those with preoperative hormonal therapy. After propensity score matching, NS did not increase the positive resection margin (RM) rate or affect PSA recurrence-free survival. Urinary QOL significantly improved in the NS group from the third month postoperatively, although no difference was found in sexual function. The RM positivity rate on the NS side was 10.8%. These findings suggest that selective NS criteria can optimize early urinary QOL without compromising oncological safety.

## 1. Introduction

Robot-assisted radical prostatectomy (RARP) is the standard surgical approach for localized prostate cancer (PCa) and offers excellent visualization and precise dissection capabilities. Nerve-sparing (NS) procedures during RARP are crucial for maintaining postoperative erectile function and urinary continence [1,2,3], in which an optimal balance between oncological control and functional outcomes is important.

Currently, there is no standardized consensus regarding the criteria for NS in RARP. Various factors have been proposed to guide decision-making, including clinical stage, prostate-specific antigen (PSA) levels, biopsy Gleason score, and magnetic resonance imaging (MRI) findings [4]. The European Association of Urology (EAU) and American Urological Association PCa guidelines recommend offering NS to patients with local PCa undergoing RARP; however, the indications are not clearly defined [5,6].

Our institution has implemented specific criteria for NS procedures, requiring the absence of Prostate Imaging-Reporting and Data System (PI-RADS) ≥3 lesions or Gleason pattern 4 or higher on biopsy in the peripheral zone (PZ). When a tumor extends through the prostatic capsule and invades the neurovascular bundle (NVB), preserving the NVB inevitably leads to a positive resection margin (RM), which in turn increases the risk of biochemical and/or local recurrence [7,8]; NS should be avoided in such cases. Against this background, our criteria were designed with the assumption that there is no significant cancer in the peripheral zone, establishing a safe margin for NS.

This study aimed to evaluate the oncological outcomes and safety of this systematic approach by retrospectively analyzing positive RM rates and PSA recurrence-free survival in patients undergoing RARP.

## 2. Materials and Methods

### 2.1. Ethics Statements

This study was approved by the Institutional Review Board of Kagawa University Faculty of Medicine (approval number: 2024-037), was conducted in accordance with the ethical principles outlined in the Declaration of Helsinki (revised in 2013), and complied with all relevant institutional and national research guidelines. Owing to the retrospective nature of this study, the requirement for informed consent was waived by the ethics committee; however, an opt-out opportunity was provided to all patients through a detailed description of the study published on our institutional website.

### 2.2. Study Design and Patients

RARP was performed using our criteria for NS indication, including PI-RADS ≥3 lesions or Gleason pattern ≥4 on biopsy in the PZ being absent. We retrospectively examined the medical records of patients who underwent RARP at the Kagawa University Hospital between August 2017 and December 2022. Patients who previously received preoperative hormonal therapy were excluded. The surgical procedure for RARP has been reported previously [9,10]. Briefly, ten surgeons performed RARP using a transperitoneal approach with the da Vinci Si^®^ robotic platform (Intuitive Surgical Inc., Sunnyvale, CA, USA). The NS procedure was primarily performed in an antegrade manner to expose a layer of intrafascial space, referencing previously reported techniques, with clipping of the NVB and, if hemostasis was required, a brief bipolar, and hemostatic suture [11]. NS layer was classified as grade 1 according to a previous report [11]. Lymph node dissection is typically performed in patients with high-risk PCa, as defined by the D’Amico risk classification; however, in May 2021, our institution revised this policy and stopped performing extended lymph node dissection.

### 2.3. Data Collection and Outcome Evaluation

Patient characteristics, including age, body mass index, PSA level at diagnosis, clinical stage, Gleason grade group, prostate volume, D’Amico risk classification, console time, blood loss, length of hospital stay, and resection margin, were obtained from the patients’ medical records. Quality of life (QOL) was assessed using the Expanded Prostate Cancer Index Composite (EPIC) and the Japanese version of the Short Form 8 (SF-8) Health Survey [12,13]. The EPIC consists of four domains (urinary, bowel, sexual, and hormonal summary scores), all of which were analyzed, except for the bowel subscale. In the SF-8, the physical and mental component summary scores were calculated using eight subscales. These scores were then transformed into a scale from 0 to 100 points using a designated scoring system, with 50 representing the average score of the general population (norm-based scoring). Higher scores indicated better QOL on both questionnaires. Paper-based questionnaires were distributed by providers in the outpatient department. Patients filled them out and returned them by mail.

### 2.4. Statistical Analysis

We excluded ineligible patients and divided them into two groups according to whether they received RARP with NS (NS and No-NS). We first compared the oncological outcomes and safety of RARP. Oncological outcomes were defined as the ratio of positive RM to PSA recurrence-free survival. PSA recurrence after RARP was defined as a PSA level >0.2 ng/mL. Safety evaluations included console time and blood loss. For comparison, the confounding factors were adjusted with propensity score matching (PSM) using age at treatment, D’Amico risk classification, prostate volume, and lymph node dissection as covariates. After a one-to-one PSM, we obtained two cohorts of 53 background-adjusted patients in the NS and No-NS groups. Second, we analyzed longitudinal QOL and continence in the entire cohort. We compared QOL based on EPIC assessments of the NS and No-NS groups at each time point. Cumulative continence rates were measured from the time of urethral catheter removal to the time of recovery from urinary incontinence. Continence was defined as achieving either pad-free status or using a single safety pad per day. Lastly, we examined the predictive factors associated with positive RM in the NS group and RM locations in the NS group. To evaluate RM, the location was divided into three parts: the apex, middle, and base (bladder side); the prostate was completely cut into slices approximately 7 mm wide on a plane perpendicular to the urethra, and two slices on the base and apex sides were cut vertically.

Continuous variables are presented as medians and interquartile ranges (IQR), and categorical variables are presented as numbers and percentages. Chi-square and Fisher’s exact tests were used for categorical variables, and the Mann–Whitney *U* test was used for continuous variables. Time-to-event was estimated using the Kaplan–Meier method and compared between groups using the log-rank test. The results of the logistic regression analyses were presented as odds ratios (ORs), 95% confidence intervals (CIs), and *p*-values. Statistical significance was set at *p* < 0.05. All statistical analyses were performed using EZR 1.54 (Saitama Medical Center, Jichi Medical University, Saitama, Japan), a graphical user interface for R (The R Foundation for Statistical Computing, Vienna, Austria) [14], and GraphPad Prism version 9.0.0 (GraphPad Software, San Diego, CA, USA).

## 3. Results

**Figure 1:**
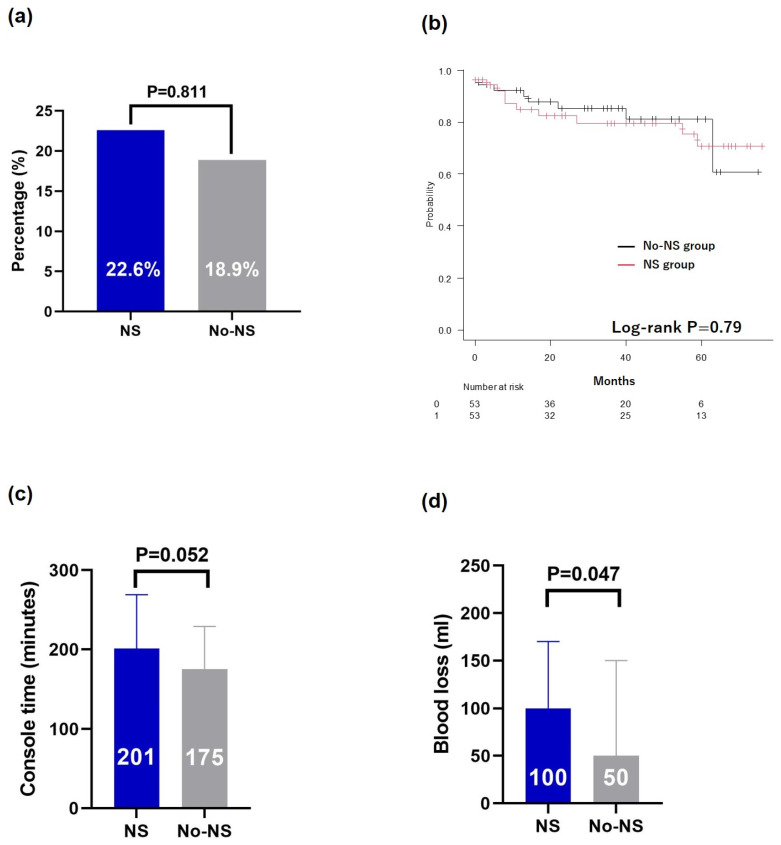
Comparison after propensity score matching. (**a**) Resection margin, (**b**) Kaplan–Meier curves of the PSA recurrence-free survival, (**c**) console time, and (**d**) blood loss. PSA, prostate-specific antigen; NS, nerve-sparing.

Among the 208 patients who underwent RARP during the study period, 20 who underwent preoperative hormonal therapy were excluded, resulting in a final cohort of 188 patients who were analyzed. The NS group comprised 68.6% of patients (*n* = 129), with 14.3% (*n* = 27) having bilateral NS and 54.3% (*n* = 102) having unilateral NS. There were statistically significant differences between the NS and No-NS groups in median age (70 vs. 72 years old, *p* = 0.023), clinical T stage (*p* < 0.001), Gleason grade group (*p* = 0.007), and D’Amico risk (*p* = 0.034) (Table 1). The median postoperative follow-up period was 39 months, with no significant differences between the groups (Table 1). After adjustment using PSM, we analyzed data from 53 patients in each group (Supplementary Table S1). There were no significant differences in positive RM rates (*p* = 0.811) (Figure 1a) or PSA recurrence-free survival (Log-rank *p* = 0.79) (Figure 1b). The console time was not significantly different between the groups (*p* = 0.052) (Figure 1c); however, blood loss was significantly higher in the NS group (*p* = 0.047) (Figure 1d).

**Figure 2:**
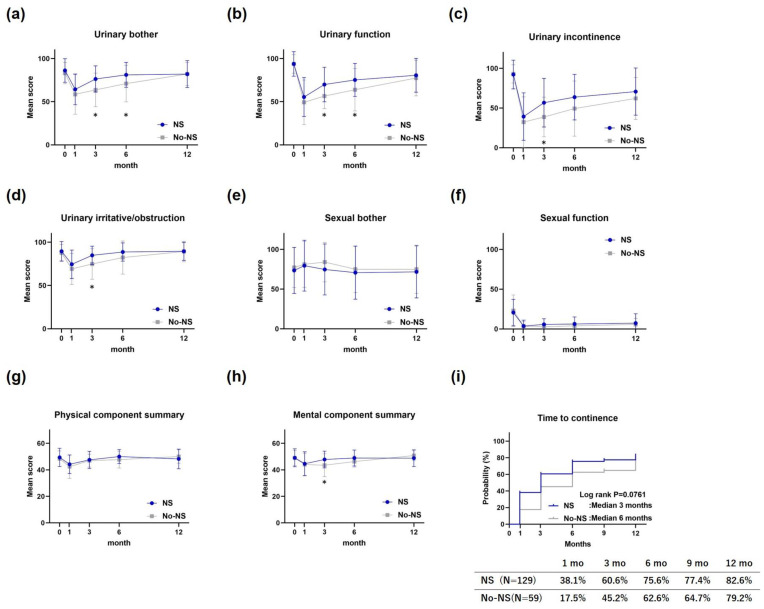
Quality of life after robot-assisted radical prostatectomy. (**a**) Urinary bother on EPIC, (**b**) urinary function on EPIC, (**c**) urinary incontinence on EPIC, (**d**) urinary irritative/obstruction on EPIC, (**e**) sexual bother on EPIC, (**f**) sexual function on EPIC, (**g**) physical component summary on SF-8, (**h**) mental component summary on SF-8, and (**i**) cumulative rates of continence. EPIC, Expanded Prostate Cancer Index; SF-8, Short Form 8 Health Survey. * indicates statistical significance.

In the EPIC urinary subscale, urinary bouts and urinary function were significantly better in the NS group than the No-NS group at 3 and 6 months postoperatively (Figure 2a,b), whereas urinary incontinence and urinary irritation/obstruction were significantly better at 3 months postoperatively (Figure 2c,d). However, at 12 months postoperatively, there were no significant differences between the NS and No-NS groups in any of the urinary subscale domains. No significant differences were observed between groups in the EPIC sexual subscale at any postoperative time point (Figure 2e,f). The SF-8 physical component summary scores showed no significant differences between the groups at any postoperative time point (Figure 2g); however, the mental component summary scores were significantly better in the NS group than in the No-NS group 3 months postoperatively (Figure 2h). The median cumulative rates of continence were 3 months postoperatively in the NS group and 6 months postoperatively in the No-NS group, which were not significantly different (log-rank test result: *p* = 0.0761) (Figure 2i).

In the NS group, the RM positivity rate was 27.9% (*n* = 36). In a multivariate logistic regression analysis using patient characteristics before RARP, diagnostic PSA and clinical T stage were predictive factors for RM positivity (OR, 1.11; 95% CI: 1.01–1.22; *p* = 0.038; OR, 1.4; 95% CI: 1.02–1.94, *p* = 0.038, respectively) (Table 2). The percentage of RM positive cases on the NS side was 10.8% (n = 14), of which 5.4% (n = 7) were base positive, 3.1% (n = 4) were mid positive, and 2.3% (n = 3) were apex positive.

## 4. Discussion

We investigated the oncological outcomes and safety of RARP with NS performed when PI-RADS ≥3 lesions or Gleason pattern ≥4 seen on biopsy in the PZ were absent. Our NS criteria did not increase the proportion of RM positives or shorten PSA recurrence-free survival compared to RARP without NS. We also found that RARP with NS had a beneficial effect on the urinary QOL in the early postoperative period. In the NS group, the risk factors for RM were the PSA level and clinical T stage. The RM positivity rate on the NS side was 10.5%. Given the lack of standardized criteria for NS, our straightforward criteria provide valuable insights into the decision-making process for NS.

After adjusting for confounding factors, our NS approach did not increase the risk of positive RMs or shorten PSA recurrence-free survival, highlighting the feasibility and clinical utility of our straightforward NS criteria. Similarly to our findings, some prior studies have reported no association between NS and the percentage of positive RMs [15,16]. However, one systematic review comparing NS and non-NS RP demonstrated a relative risk of 1.5 for side-specific positive RMs, although no effect of NS on biochemical recurrence was observed [17]. A previous study reported that the percentage of positive RMs on the NS side was 9% [18]. However, when interpreting these findings, it is essential to consider the possibility of selection bias in NS indication and differences in follow-up duration across studies. RARP with NS on the side with extraprostatic extension, defined as clinical T3 or higher, carries a potentially higher risk of positive RM. Because of this, nomograms predicting extraprostatic extension can serve as valuable tools for determining the indications for NS. Previous studies have reported that such nomograms typically incorporate factors such as PSA level, clinical T stage, biopsy Gleason score, and MRI findings [4,19]. Our NS criteria include these factors, which are consistent with existing evidence.

In our study, the positive RM rate on the NS side was 10.8%, with approximately half of the cases occurring at the base of the prostate. Multivariate analysis of RM positivity on the NS side suggested an association between elevated PSA levels and a higher clinical T stage. A detailed analysis of NS-side cases in the NS group revealed that more than 70% of the patients had findings in the transition zone on MRI or biopsy (Supplementary Table S2). Even in cases where no abnormalities are detected in the peripheral zone on MRI or biopsy, caution is warranted in patients presenting with these risk factors. Certain techniques may be helpful for safe treatment using RARP with NS. Intraoperative neurovascular structure–adjacent frozen section examination (NeuroSAFE) involves evaluation of the RM of the prostate adjacent to the NVB during radical prostatectomy [20]. Using the NeuroSAFE technique can decrease the percentage of positive RMs [21]. In our NS criteria-based RARP approach, techniques such as NeuroSAFE may help reduce the rate of positive RMs on the NS side in patients at a higher risk of RM positivity. Accurate localization of cancer is crucial for RARP with NS. Next-generation imaging modalities, such as prostate-specific membrane antigen positron emission tomography-computed tomography (PSMA PET-CT), have the potential to improve tumor localization. Indeed, PSMA PET-CT has been shown to be particularly effective at detecting higher-grade PCa and allows more accurate local staging of the prostate compared to conventional imaging with CT or bone scintigraphy [22]. Additionally, it has been reported to significantly predict PSA recurrence-free survival following RP [23]. Similarly, reports have described the use of three-dimensional prostate MRI models and augmented reality (AR) in RARP with NS to reduce the percentages of positive RMs [24,25,26,27]. As an extension of AR technology, the utility of an automatic AR system guided by artificial intelligence at identifying tumor location at the level of the preserved NVB at the completion of RARP with NS has been investigated [28]. One recent study demonstrated that at the end of the extirpative phase, an automatic AR artificial intelligence-driven system utilizing a virtual 3D prostate model enabled precise localization of the tumor at the level of the preserved NVB and facilitated a selective excisional biopsy while preserving the remaining portion of the bundle. This technology successfully identified the lesion on the NVB in 87.5% of patients with pT3 disease and was reported to maintain oncological safety without increasing the percentage of positive RMs.

In terms of QOL, RARP with NS demonstrated benefits for urinary continence, but did not contribute to improvements in sexual function. It significantly enhanced early urinary QOL and was associated with improved mental QOL. Our results also suggested the potential contribution of NS to a shorter time to achieve continence, which was not statistically significant (Figure 2i). The observed benefits of early urinary continence are consistent with a previous report [29]. Although differences in QOL between the groups were no longer evident at 12 months postoperatively, earlier improvement in QOL following RARP was clinically meaningful. The mechanism underlying the link between NS and urinary continence recovery remains unclear. Some anatomical studies have reported the supply of intrapelvic somatic nerves to the striated urethral sphincter [30], indicating that urinary continence recovery may be explained by the preservation of these nerves [29]. Alternatively, one study proposed that continence improvement is primarily attributed to the excision techniques used during NS surgery, rather than the preservation of the NVB itself [31]. No significant differences were observed between the NS and No-NS groups in terms of sexual function or discomfort at each postoperative point (Figure 2e,f). In the NS group, the lack of improvement in sexual function suggests that the patients did not perceive a significantly greater degree of worry. A previous study on Japanese cohorts undergoing RARP reported higher rates of bother-improvement than postoperative sexual function recovery [32]. Although the mechanism underlying these observations remains unclear, Japanese patients may place relatively less emphasis on sexual function as a key aspect of postoperative recovery.

The present study had some limitations. First, the study was conducted at a single institution with a relatively small sample size, which limits the generalizability of the findings, and further studies with larger cohorts are required for validation. Second, the observation period was relatively short (approximately 3 years), necessitating the use of PSA recurrence-free survival as a surrogate endpoint for long-term outcomes. Third, RARP was performed by ten different surgeons, as our institution serves as an educational center. The quality of NS procedures may also vary owing to the involvement of multiple surgeons; however, variations in the surgical technique were minimized through consistent supervision by a single specialist, ensuring procedural standardization. Fourth, our analyzed cohort included referred patients rather than those from a single institution; therefore, the indications and methods for prostate biopsy, including PI-RADS-based targeting, lacked uniformity.

## 5. Conclusions

We demonstrated that nerve-sparing criteria based on the absence of PI-RADS ≥3 lesions or Gleason pattern ≥4 in the PZ provided acceptable oncological outcomes without increasing positive surgical margin rates. Although this approach is associated with improved early postoperative urinary QOL, careful patient selection remains crucial, particularly in those with elevated PSA levels and a higher clinical T stage. Our results suggest that this systematic approach for patient selection for NS can achieve a favorable balance between oncological control and functional outcomes in RARP.

## Figures and Tables

**Table 1:** Patients’ backgrounds.

Variables	Nerve-Sparing Group	No-Nerve-Sparing Group	*p*-Value
Patients	129	59	
Age, median (IQR)	70 (66–74)	72 (67.5–76)	0.023
BMI, kg/m^2^, median (IQR)	22.9 (21.6–25.1)	23.7 (21.8–25.8)	0.264
PSA at diagnosis, ng/mL median (IQR)	6.38 (4.78–9.02)	6.80 (5.30–9.88)	0.288
Clinical stage, n (%)			<0.001
cT1c	15 (11.6)	5 (5.1)	
cT2a	90 (69.8)	31 (52.5)	
cT2b	3 (2.3)	8 (13.6)	
cT2c	3 (2.3)	9 (15.3)	
cT3a	15 (11.6)	6 (10.2)	
cT3b	3 (2.3)	2 (3.4)	
Gleason grade group, n (%)			0.007
1	24 (18.6)	1(1.7)	
2	33 (25.6)	24(40.7)	
3	37 (28.7)	16(27.1)	
4	25 (19.4)	12(20.3)	
5	10 (7.8)	6 (10.2)	
D’Amico risk, n (%)			0.034
Low	15 (11.6)	1 (1.7)	
Intermediate	64 (49.6)	28 (47.5)	
High	50 (38.8)	30 (50.8)	
Prostate volume, mL, median (IQR)	28.26 (22–36)	26 (19.5–32)	0.087
Lymph node dissection, n (%)	42 (32.6)	30 (50.8)	0.023
Follow-up period, month, median (IQR)	39 (22–56)	39 (23–48)	0.852

IQR, interquartile range; BMI, body mass index.

**Table 2:** Multivariate logistic regression analysis of the predictive factors for resection margin positivity.

	Odds Ratio	95% CI	*p* Value
PSA at diagnosis	1.110	1.010, 1.220	0.038
Gleason grade group at biopsy	1.120	0.792, 1.580	0.526
cT stage	1.400	1.020, 1.940	0.038
Percentage of biopsy positive core	0.997	0.983, 1.010	0.680

CI, confidence interval.

## Data Availability

The datasets generated and analyzed during the current study are not publicly available owing to our hospital policy but are available from the corresponding author upon reasonable request.

## Supplementary Materials

The following supporting information can be downloaded at: https://www.mdpi.com/article/10.3390/cancers17060962/s1, Table S1: Patients’ backgrounds after propensity score matching; Table S2: Details of nerve-sparing side resection margin positive cases in nerve-sparing group.

## Abbreviations

The following abbreviations are used in this manuscript:

NS	nerve-sparing
RARP	robot-assisted radical prostatectomy
PI-RADS	Prostate Imaging-Reporting and Data System
PZ	peripheral zone
PSM	propensity score matching
RM	resection margin
PSA	prostate-specific antigen
QOL	quality of life
OR	odds ratio
PCa	prostate cancer
MRI	magnetic resonance imaging
EAU	European Association of Urology
NVB	neurovascular bundle
EPIC	Expanded Prostate Cancer Index Composite
SF-8	Short Form 8
IQR	interquartile range
CI	confidence interval
NeuroSAFE	neurovascular structure–adjacent frozen section examination
