# Supplementary material for: Nerve-Sparing Robotic-Assisted Radical Prostatectomy Based on the Absence of Prostate Imaging-Reporting and Data System ≥3 or Biopsy Gleason Pattern ≥4 in the Peripheral Zone
Source: Cancers (Basel). 2025 Mar 12;17(6):962. doi: 10.3390/cancers17060962 (PMC11940342; doi:10.3390/cancers17060962)
Supplement: Supplementary file 1 [file cancers-17-00962-s001.zip › Supplementary Table S1.pdf]

Supplementary Table S1. Patients' backgrounds after propensity score matching.

Variables	NS group	No-NS group	p-Value
Patients	53	53	
Age, median (IQR)	70 (67-70)	72 (67.5-76)	0.213
D'Amico risk, n (%)			
Low	5 (9.4)	1 (1.9)	0.199
Intermediate	22 (41.5)	28 (52.8)	
High	26 (49.1)	24 (45.3)	
Prostate volume, ml, median (IQR)	28 (23-31)	26 (20-33)	0.633
Lymph node dissection, n (%)	30 (43.4)	19 (54.7)	0.331

IQR, interquartile range
