# Supplementary material for: Nerve-Sparing Robotic-Assisted Radical Prostatectomy Based on the Absence of Prostate Imaging-Reporting and Data System ≥3 or Biopsy Gleason Pattern ≥4 in the Peripheral Zone
Source: Cancers (Basel). 2025 Mar 12;17(6):962. doi: 10.3390/cancers17060962 (PMC11940342; doi:10.3390/cancers17060962)
Supplement: Supplementary file 1 [file cancers-17-00962-s001.zip › Supplementary Table S2.pdf]

Supplementary Table S2. Details of nerve-sparing side resection margin positive cases in nerve-sparing group.

	Transition Zone		Peripheral Zone
	MRI PIRADS	Gleason Score	Gleason Score
Apex	0	0	0
Apex	0	3+3	0
Apex	0	0	3+3
Mid	3	4+5	0
Mid	0	3+3	0
Mid	3	3+3	0
Mid	0	0	0
Base	5	3+3	0
Base	0	3+4	0
Base	3	3+4	0
Base	5	4+3	0
Base	5	4+4	0
Base	0	0	3+3
Base	5	3+3	0

PI-RADS, Prostate Imaging-Reporting and Data System
